# Characterization of the gut virome in patients with nonalcoholic fatty liver disease

**DOI:** 10.1186/s12967-025-07443-w

**Published:** 2025-11-28

**Authors:** Lvyue Wang, Leyi Wang, Min Liu, Qi Yuan, Lin Cheng, Huixiang Chen, Shanliang Mao, Shenghui Li, Qiulong Yan, Guorui Xing, Ning Zheng

**Affiliations:** 1 Department of Critical Care Medicine, The Second Hospital of Nanjing, Affiliated to Nanjing University of Chinese Medicine, Nanjing, China; 2 Department of Gastroenterology, Loudi Central Hospital, Loudi, China; 3 Department of Biochemistry and Molecular Biology, College of Basic Medical Sciences, Dalian Medical University, Dalian, China; 4 Puensum Genetech Institute, Wuhan, China; 5 Department of Pediatrics, Loudi Health Center for Women and Children, Loudi, China; 6 The Fifth Affiliated Hospital of Southern Medical University, Guangzhou, China

**Keywords:** Nonalcoholic fatty liver disease, Gut virome, Metagenomics, Serum metabolomics

## Abstract

**Background:**

Nonalcoholic fatty liver disease (NAFLD) is a prevalent metabolic disorder with complex gut microbiome involvement. While bacterial dysbiosis in NAFLD has been widely studied, the role of the gut virome remains largely unexplored.

**Methods:**

We profiled gut viral communities from 90 NAFLD patients and 90 non-NAFLD controls using whole-metagenome shotgun sequencing. Viral taxonomic composition, host associations, and functional gene repertoires were analyzed. Serum metabolomic data were integrated to assess virus–metabolite interactions, and random forest models were constructed to evaluate the diagnostic potential of viral signatures.

**Results:**

Overall viral diversity showed no significant differences between NAFLD and controls, but subtle compositional shifts were detected at the vOTU level, with 105 viruses enriched in NAFLD and 185 in non-NAFLD individuals. NAFLD-enriched phages primarily targeted Bacteroides, whereas non-NAFLD-enriched phages were associated with beneficial genera such as Faecalibacterium, Oscillibacter, and Prevotella. Functional annotation revealed a reorganization of viral gene repertoires: genes involved in DNA recombination and horizontal transfer (e.g. int, recD) were depleted, while those related to host interaction and stress response (e.g. xerD, dnaK, hipB) were enriched in NAFLD, indicating enhanced viral persistence and host communication. Serum metabolomic profiling identified 8 differential metabolites, and correlation analysis linked specific vOTUs with altered metabolic pathways. A random forest model based on viral features achieved an AUC of 0.758, outperforming the bacterial model, while integration of viral and bacterial features further improved prediction (AUC = 0.837).

**Conclusion:**

The gut virome in NAFLD undergoes compositional and functional remodeling characterized by a shift toward host-adaptive, metabolically interactive viral communities. These viral alterations are closely associated with host metabolic changes and demonstrate strong diagnostic potential. Our findings highlight the virome as an overlooked yet critical component of the gut ecosystem in NAFLD pathogenesis and as a promising source of noninvasive biomarkers for disease prediction and monitoring.

**Supplementary Information:**

The online version contains supplementary material available at 10.1186/s12967-025-07443-w.

## Introduction

The human gut virome, comprising both bacteriophages and eukaryotic viruses, plays a crucial role in maintaining intestinal homeostasis and shaping microbial community dynamics [1]. Viral communities modulate bacterial populations through predation, horizontal gene transfer, and immune regulation, thereby influencing host metabolism and disease susceptibility [2]. Increasing evidence links virome alterations to inflammatory bowel disease, colorectal cancer, and metabolic disorders, yet the gut virome remains far less characterized than the bacterial microbiome, particularly in chronic liver diseases such as nonalcoholic fatty liver disease (NAFLD) [3].

NAFLD encompasses a spectrum of conditions ranging from simple steatosis (NAFL) to nonalcoholic steatohepatitis (NASH), which can progress to fibrosis, cirrhosis, and hepatocellular carcinoma [4]. Closely associated with obesity and type 2 diabetes, NAFLD has become a major global health concern, with its prevalence in Asia now comparable to Western countries [5]. A major clinical challenge in NAFLD is the absence of overt symptoms in its early stages. The liver, often referred to as a “silent” organ, may sustain substantial damage before clinical signs become apparent, resulting in delayed diagnosis and disease progression to advanced stages such as cirrhosis [6]. This silent course, coupled with lifestyle changes and rising metabolic syndrome prevalence, has contributed to a global increase in NAFLD incidence. Although bacterial dysbiosis and associated metabolites have been implicated in NAFLD pathogenesis through oxidative stress, inflammation, and endotoxin-mediated injury [7–10], the contribution of the gut virome remains largely unexplored.

Recent evidence suggests that viruses can shape bacterial ecology and metabolic potential, thereby indirectly influencing liver disease progression [11]. However, current research has focused mainly on bacteria, while the gut virome remains understudied. Viral communities influence bacterial dynamics and disease development [12], yet existing human virome studies in NAFLD are limited by outdated viral reference databases and rarely integrate metabolomic data, thereby limiting the functional and mechanistic interpretation of virome–host metabolic interactions [13, 14]. Meanwhile, cross-kingdom interactions between viruses and bacteria, which may jointly affect host metabolism, are largely overlooked.

To address these critical gaps, we conducted a comprehensive investigation of gut virome alterations in a rigorously matched cohort of 90 NAFLD patients and 90 non-NAFLD individuals using metagenomic sequencing and serum metabolomics [15]. By integrating viral, bacterial, and metabolic profiles, this study aims to uncover novel mechanisms linking the gut virome to liver disease pathogenesis and to identify potential viral biomarkers for diagnosis and therapeutic targeting.

## Materials and methods

### Data source

All fecal metagenomic sequencing data analyzed in this study were obtained from publicly available datasets in the NCBI Sequence Read Archive (SRA). A total of 180 samples were included, comprising 90 patients with NAFLD and 90 non-NAFLD controls. Among them, 176 samples were derived from project PRJNA728908, while four additional publicly available control samples (SRR13279648, SRR13279753, SRR13279664, and SRR13279666) were obtained from PRJNA686835 [15]. These datasets were generated using comparable sequencing platforms and preprocessing pipelines, allowing for consistent integration in downstream analyses. These datasets were originally derived from a nested case–control study within a prospective, community-based cohort of ~2,500 Han Chinese adults enrolled in 2014. NAFLD diagnosis was made at the 2018 follow-up visit via abdominal ultrasonography, the first-line diagnostic method endorsed by the Asian Pacific Association for the Study of the Liver (APASL). The NAFLD group (*n* = 90) comprised individuals who developed NAFLD between baseline and follow-up, while the control group (*n* = 90) consisted of participants who remained disease-free. Participants with T2DM, metabolic syndrome, hypertension, or recent use of antibiotics or antifungal agents were excluded according to the original study protocol. While individual-level metadata were not publicly available, the source publication reported no significant differences in baseline clinical variables such as liver enzymes, insulin resistance, lipid profiles, or inflammatory markers. Additionally, serum metabolomic profiles from the same 180 individuals were downloaded from the MetaboLights database under accession ID MTBLS2615, which were generated using standardized metabolomics workflows. These were used for subsequent multi-omics integration (Table S1).

### Metagenomic quality control and preprocessing

Quality control of metagenomic reads was performed using fastp [16], which included: (1) trimming of polyG tails, (2) removal of reads < 90 bp, (3) filtering reads with a mean Phred score < 20, (4) discarding reads with > 30% low-quality bases (Phred < 20), (5) excluding reads with complexity < 30%, and (6) removing unpaired reads. These were used for subsequent multi-omics integration. Raw reads were preprocessed using fastp software (v0.20.1) [16] with the following parameters: -l90 -q20 -u30 -y –trim_poly_g, to eliminate low-quality sequences. Human reads were removed from the high-quality reads using Bowtie2 [17] alignment to the human reference genome (GRCh38).

### Gut bacteriome and virome profiling

The gut bacterial composition was profiled from fecal metagenomic data using MetaPhlAn4 [18], which performs taxonomic classification based on clade-specific marker genes. Relative abundances were computed at the species level and subsequently aggregated to genus and phylum levels by summing the abundances of constituent species within each sample.

For viral profiling, we employed the Chinese Gut Viral Catalog (cnGVC) [19], a comprehensive reference database consisting of 67,159 non-redundant viral operational taxonomic units (vOTUs) reconstructed from 10,324 publicly available fecal metagenomes. High-quality metagenomic reads were mapped to the cnGVC using Bowtie2 with a 95% nucleotide identity cutoff to assign species-level viral taxonomy [20]. To account for genome size variability, viral abundance was normalized by genome length. Only vOTUs with ≥10% genome coverage (i.e., reads mapped to at least 10% of the viral genome) were retained for downstream analysis [21]. Relative abundances of individual vOTUs were calculated by normalizing the number of reads mapped to each vOTU against the total number of mapped reads per sample. To generate family-level viral profiles, normalized abundances of vOTUs belonging to the same family were aggregated. Taxonomic annotation followed the latest classification criteria established by the International Committee on Taxonomy of Viruses (ICTV, https://ictv.global/). The eukaryotic and prokaryotic viruses within vOTUs were characterized using IPEV, a novel approach that integrates trinucleotide pairwise relative distance and frequency with a two-dimensional convolutional neural network for distinguishing between prokaryotic and eukaryotic viruses in viromes [22].

### Functional analysis of NAFLD-related vOTUs

We performed functional annotation of viral proteins using the DIAMOND [23] with the parameters “--query-cover 50 --subject-cover 50 --evalue 1e-5 --min-score 50 --max-target-seqs 50” against the Kyoto Encyclopedia of Genes and Genomes (KEGG) database [24]. Each protein was assigned a KO (KEGG Ortholog) identifier based on the best hit in the database.

The incidence of each KO within a group was calculated as the number of vOTUs carrying that KO divided by the total number of vOTUs in the group. Fisher’s exact test (implemented via the *fisher.test* function) was applied to determine whether the incidence of each KO differed significantly between groups. KOs with a p-value < 0.05 were considered significantly differentially present.

### Statistical analyses

All statistical analyses in this study were conducted on the R (v4.2.1) platform. Principal coordinates analysis (PCoA) of the Bray-Curtis distance was carried out and visualized using the *vegan* package [25]. Permutational multivariate analysis of variance (PERMANOVA) was realized with the adonis function of the *vegan* package, with an adonis p-value generated from 1,000 permutations. We employed the Wilcoxon rank-sum test to assess statistical differences in the diversity levels between the two cohorts. MaAsLin2 analysis with strict multiple-testing correction (*p* < 0.05) was subsequently applied to identify differentially abundant features after adjusting for gender covariates in datasets containing complete metadata. Results with p-value less than 0.05 were considered statistically significant. Spearman correlation analysis was performed to assess associations between vOTUs and metabolite biomarkers, and the resulting p-values were adjusted for multiple testing using the Benjamini–Hochberg (BH) method. Correlation networks and plots were visualized using the *ggplot2* package in R. Random forest models were trained using the *randomForest* package (1,000 trees) to distinguish NAFLD patients and non-NAFLD based on the abundance profiles of the differential viral signatures.

## Results

### Clinical characteristics of the study cohort

The study included 90 NAFLD patients and 90 non-NAFLD controls. The clinical characteristics of the participants are summarized in Table S1. No significant differences were observed between the two groups in terms of age, sex distribution, or BMI. Detailed demographic and clinical data can be found in the previously published study [15].

### Structural and diversity changes in the gut virome of NAFLD patients

**Fig. 1:**
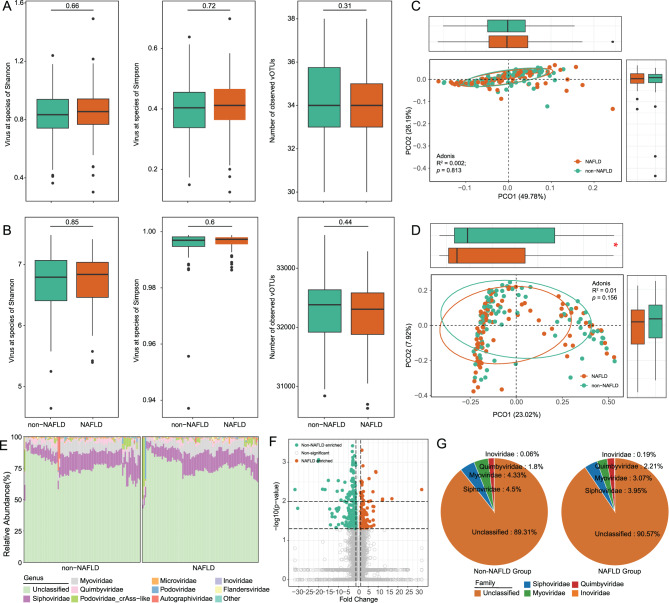
Comparison of gut virome diversity, composition, and differential features between NAFLD and non-NAFLD individuals. (A, B). Boxplots and scatterplots showing within-sample viral richness and diversity at the vOTUs level (**a**) and family level (**b**), based on observed counts, Shannon index, and Simpson index. (**c, d**) Principal coordinates analysis (PCoA) based on bray–curtis dissimilarity of viral profiles at the family (**c**) and vOTUs (**d**) levels. Ellipses represent 95% confidence intervals for each group. Marginal boxplots indicate sample scores along the PCoA1 and PCoA2 axes. (**e**) Bar plot showing the relative abundance of the top 10 most prevalent viral families across all samples. (**f**) V volcano plot of differentially abundant vOTUs between groups. Orange dots represent vOTUs enriched in NAFLD, and green dots represent those enriched in non-NAFLD. (**g**) Pie charts showing the taxonomic distribution of differentially abundant vOTUs at the viral family level in NAFLD and non-NAFLD groups

To investigate gut virome alterations in NAFLD, we first assessed within-sample viral diversity and richness. At the family level, NAFLD patients exhibited a slight, non-significant increase in diversity and a marginal decrease in the number of observed families (*p* > 0.05, Fig. 1A). Similar patterns were observed at the vOTU level, with minor changes in diversity and richness (Fig. 1B). We next examined overall virome composition using PCoA based on Bray–Curtis dissimilarity. No significant differences were observed between NAFLD patients and controls at the family level (PERMANOVA: R^2^ = 0.002, *p* = 0.813, Fig. 1C). However, at the vOTU level, PERMANOVA revealed a significant group separation along one principal coordinate (*p* < 0.05, Fig. 1D), indicating subtle but detectable shifts in viral community structure.

To further characterize viral community composition, all detected viruses were categorized into prokaryotic and eukaryotic groups. In NAFLD patients, prokaryotic viruses predominated, accounting for 96.56% of the total viral population, whereas only 3.44% were eukaryotic viruses. We then compared α- and β-diversity between NAFLD and control groups within each viral category (Fig. S1). Neither Shannon index nor the number of observed eukaryotic or prokaryotic viruses showed significant differences between groups (*p* > 0.05). Consistently, PCoA analysis based on Bray–Curtis distances revealed no significant separation in either eukaryotic (R^2^ = 0.005, *p* = 0.389, Fig. S1) or prokaryotic (R^2^ = 0.007, *p* = 0.181) viral communities, suggesting that the overall viral composition remained relatively stable in NAFLD.

To further explore compositional changes, we analyzed viral taxonomic profiles. Both groups were dominated by *Siphoviridae*, *Myoviridae*, *Quimbyviridae*, and *crAss-like Podoviridae*. To account for potential gender-related confounding, we performed multivariable association analysis using MaAsLin2, which revealed no significant differences at the family level (fold change > 1.2, *p* < 0.05, prevalence > 10%). However, *Microviridae* showed a significant association between groups, although the change did not reach the predefined fold change threshold of 1.2. At the vOTU level, differential abundance analysis identified 185 vOTUs enriched in non-NAFLD individuals and 105 enriched in NAFLD patients (Fig. 1F, Table S2). Among the classified vOTUs, *Siphoviridae* was the most represented family, followed by *Myoviridae* and *Quimbyviridae*, suggesting that specific phage taxa may contribute to virome alterations associated with NAFLD (Fig. 1G).

### Correlations between gut virome and serum metabolome

**Fig. 2:**
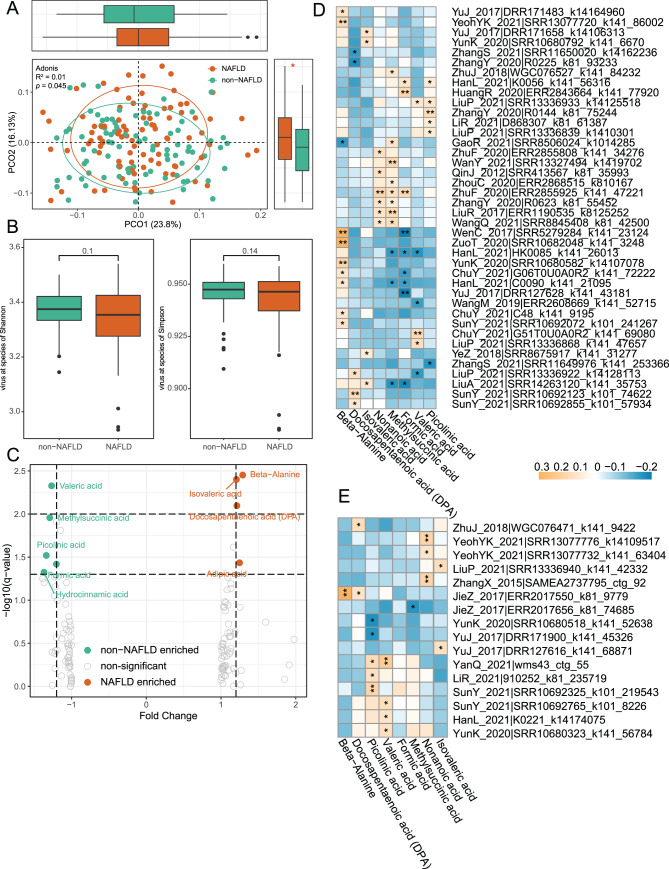
Interaction between the gut virome and host serum metabolomes. (**a**) boxplots showing α-diversity of serum metabolites, measured by Shannon and Simpson indices, in NAFLD patients and non-NAFLD controls. (**b**) Principal coordinates analysis (PCoA) based on bray–curtis distances illustrating overall differences in metabolomic profiles between the two groups. (**c**) Volcano plot showing differentially abundant metabolites. Orange dots indicate metabolites significantly enriched in the NAFLD group, while green dots represent those enriched in the non-NAFLD group (**d, e**) Heatmaps showing Spearman’s rank correlations between differential viral operational taxonomic units (vOTUs) and serum metabolites. Panel (**d**) shows correlations between NAFLD-enriched vOTUs and differential metabolites, whereas panel (**e**) shows correlations between NAFLD-depleted vOTUs and differential metabolites. p-values were adjusted using the benjamini–Hochberg method, *p < 0.05, **p < 0.01

To explore the potential implications of gut virome alterations, we integrated viral and serum metabolomic data to examine associations between viral community composition and host metabolic profiles. While no significant differences in metabolite α-diversity (Shannon and Simpson indices) were observed between groups (Fig. 2A), β-diversity analysis revealed significant separation based on PCoA (*p* < 0.05, Fig. 2B).

Wilcoxon rank-sum tests identified eight significantly differential metabolites (fold change > 1.2, *p* < 0.05, Fig. 2C, Table S3). Metabolites enriched in NAFLD included beta-alanine, isovaleric acid, and docosapentaenoic acid (DPA), whereas nonanoic acid, picolinic acid, formic acid, valeric acid, and methylsuccinic acid were enriched in non-NAFLD individuals. Correlation analysis further revealed significant associations between these metabolites and specific viral taxa: 16 non-NAFLD-enriched vOTUs were linked to non-NAFLD-enriched metabolites (Fig. 2D), while 40 NAFLD-enriched vOTUs were associated with metabolites elevated in NAFLD (Fig. 2E, q < 0.05). These findings suggest that gut virome alterations may influence host metabolic phenotypes through specific virus–metabolite interactions.

### NAFLD-associated gut functional gene signatures

**Fig. 3:**
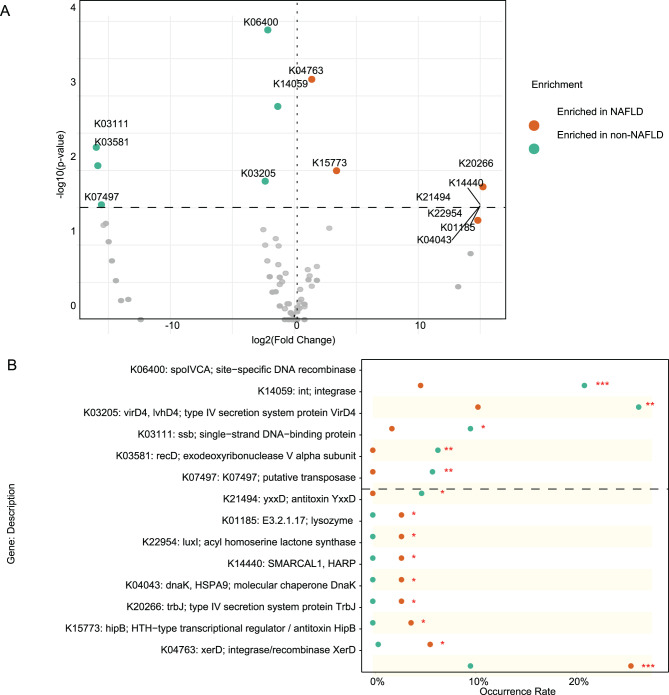
Overview of functional and taxonomic differences in the gut virome between NAFLD and non-NAFLD individuals. (**a**) Volcano plot displaying the Fold changes and p-values of all vOTUs in the comparison between NAFLD and non-NAFLD individuals. (**b**) Occurrence rates of 14 differential viral functions identified in NAFLD -enriched and NAFLD-depleted vOTUs. Statistical significance was determined using Fisher’s exact test, with *p* < 0.05 considered significant

Functional annotation revealed distinct differences in viral-encoded genes between NAFLD and non-NAFLD individuals. In total, 14 KEGG orthologs (KOs) showed significant differences in carriage frequency among the 290 NAFLD-associated vOTUs (Fisher’s exact test, adjusted p-value < 0.05; Fig. 3A, Table S4). Based on Fisher’s exact test, several viral functional genes exhibited significant differences in occurrence frequency between NAFLD-enriched and NAFLD-depleted viruses. Functions related to DNA replication and recombination, including *putative transposase (K07497)*, *exodeoxyribonuclease V alpha subunit (recD, K03581)*, *single-strand DNA-binding protein (ssb, K03111)*, *type IV secretion system protein VirD4 (K03205)*, *integrase (int, K14059)*, and *site-specific DNA recombinase (spoIVCA, K06400)*, showed reduced prevalence among NAFLD-associated viruses, suggesting a potential decline in viral genomic mobility and horizontal gene transfer activity (Fig. 3B).

In contrast, genes involved in stress response, transcriptional regulation, and host interaction, including *integrase/recombinase XerD (K04763)*, *HTH-type transcriptional regulator or antitoxin HipB (K15773)*, *type IV secretion system protein TrbJ (K20266)*, *molecular chaperone DnaK (K04043)*, *chromatin remodeling protein SMARCAL1 (K14440)*, *acyl homoserine lactone synthase LuxI (K22954)*, *lysozyme (K01185)*, and *antitoxin YxxD (K21494)*, were significantly enriched in NAFLD-enriched viral genomes. These upregulated functions indicate an enhanced potential for virus–host communication, stress adaptation, and lysogenic regulation, reflecting a functional shift toward more active host–virus interactions in the gut virome of NAFLD patients.

### Host specificity of NAFLD-linked gut virome

**Fig. 4:**
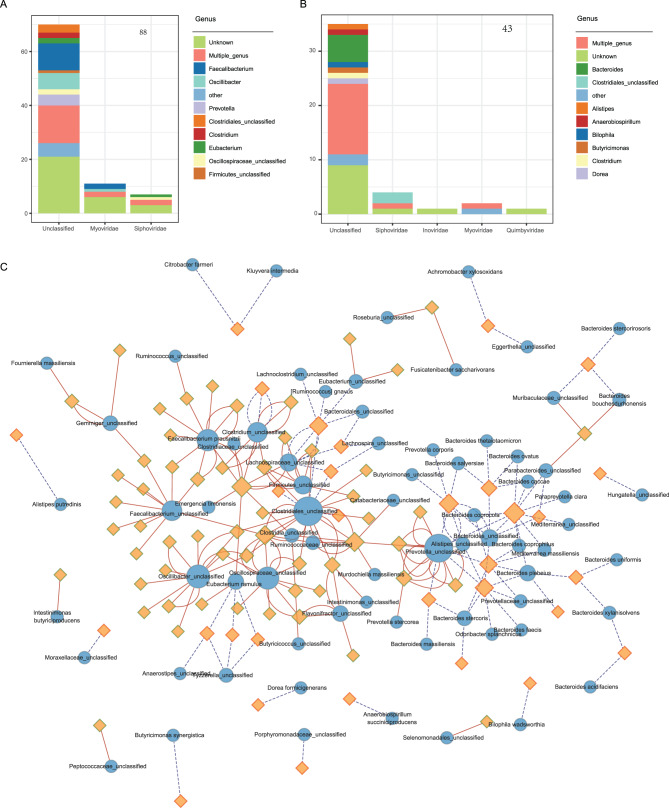
Predicted virus–host interactions network in NAFLD. (**A**, **B**) stacked bar plots showing the taxonomic classification and predicted prokaryotic host genera of vOTUs enriched in the NAFLD group (**a**) and non-NAFLD group (**b**). vOTUs predicted to infect multiple bacterial genera are labeled as “multiple-genus.” (**c**) Network visualization of NAFLD-associated vOTUs and their predicted bacterial hosts. Nodes represent vOTUs (rhombuses) and host bacterial species (circles), with border colors indicating enrichment direction (red for NAFLD-enriched, green for non-NAFLD-enriched). Edges indicate predicted virus–host associations: red solid lines represent associations in the non-NAFLD group, blue dotted lines represent associations in the NAFLD group

Viral functions are often carried out in conjunction with their bacterial hosts. To better understand the ecological context of NAFLD-associated viruses, we investigated the predicted hosts of differentially abundant vOTUs. Among the 105 viruses enriched in the NAFLD group, only 43 were identified as bacteriophages (Fig. 4A), with predicted hosts primarily belonging to *Bacteroides*, *Clostridiales_unclassified*, *Alistipes*, and related taxa. In contrast, among the 185 viruses enriched in the non-NAFLD group, 88 were bacteriophages (Fig. 4B), predominantly targeting *Faecalibacterium*, *Oscillibacter*, *Prevotella*, and other commensal genera.

A host–virus interaction network (Fig. 4C) further illustrated these differences. *Faecalibacterium prausnitzii*, a known beneficial commensal, was linked to numerous viruses enriched in the non-NAFLD group. Similar associations were observed for members of *Oscillospiraceae*, *Clostridiales*, and *Prevotella*. In contrast, NAFLD-enriched viruses were more frequently associated with *Bacteroides*, suggesting that they may influence host metabolism or immune responses in ways that promote NAFLD pathogenesis. However, further mechanistic studies are required to confirm these functional roles.

### Predicting NAFLD status using gut virome and bacteriome signatures

**Fig. 5:**
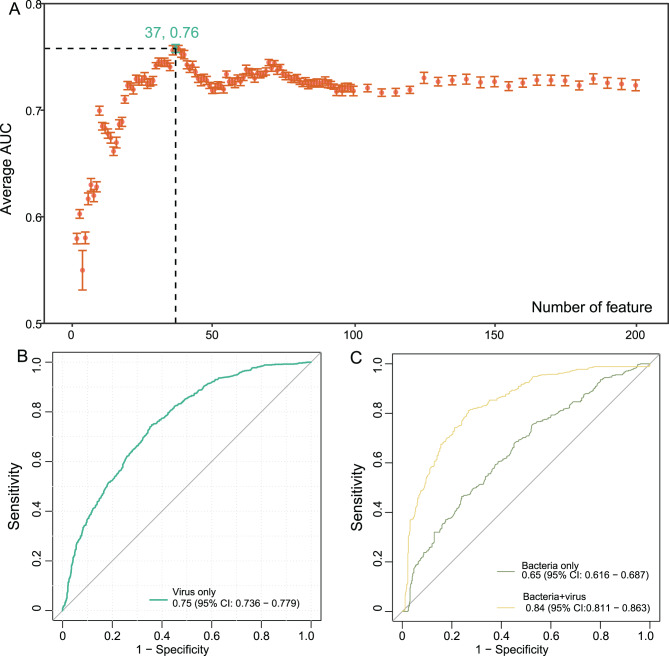
Predictive performance of gut viral and bacterial signatures for NAFLD classification. (**a**) Line chart showing the average area under the curve (AUC) values of random forest models trained with increasing numbers of top-ranked NAFLD-associated viral operational taxonomic units (vOTUs). (**b**) Receiver operating characteristic (ROC) curve showing the performance of the random forest model based solely on viral feature. (**c**) ROC curves comparing the predictive performance of bacterial features alone and the combined model combining both viral and bacterial features

Finally, we evaluated the predictive potential of gut virome signatures for distinguishing NAFLD status. Using the differentially abundant vOTUs as input features, a random forest classifier achieved an overall predictive performance with an AUC exceeding 0.70 across repeated cross-validations. Notably, when the top 37 vOTUs ranked by importance were used for modeling, the predictive performance reached its highest value (AUC = 0.76; Fig. 5A–B).

To compare the predictive capacity of bacterial features, we identified 28 differential bacterial species using the same filtering criteria applied to viruses (MaAsLin2, fold change > 1.2, *p* < 0.05, prevalence > 10%). A random forest model constructed based on these bacterial features yielded a lower predictive accuracy (AUC = 0.65; Fig. 5C), suggesting that viral signatures may serve as more robust and informative biomarkers for NAFLD classification. Furthermore, when bacterial and viral features were combined in an integrated model, the predictive performance substantially improved (AUC = 0.84; Fig. 5C). These findings indicate that bacterial and viral components provide complementary information, and their integration can enhance disease prediction, highlighting the potential of cross-domain microbial signatures as noninvasive biomarkers for NAFLD.

## Discussion

Numerous studies have revealed that there is a disturbance in the intestinal microbiota in patients with NAFLD, mainly focusing on the analysis of the intestinal bacterial communities [26–28]. However, studies on the relationship between intestinal viral communities and NAFLD are relatively scarce, and the aspects of metabolomics as well as viral and host factors have been overlooked [13, 14]. Here, we conducted a study on the intestinal viral communities of a group including 90 non-NAFLD individuals and 90 NAFLD patients [15]. At the same time, we also investigated the relationship between the types of intestinal viruses and the host serum metabolites, emphasizing the potential impact of the types of intestinal viruses on NAFLD patients. Finally, based on the characteristics of the intestinal viral communities, we used random forests to predict the disease for NAFLD patients and established potential biomarkers [29].

Our virome analysis indicated no statistically significant differences in the diversity or overall structure of viral communities between patients with NAFLD and the non-NAFLD group. Although these values do not meet the conventional threshold for statistical significance (*p* < 0.05), a mild trend suggests that differences in viral communities among NAFLD patients may be subtle or confined to specific viral taxa, rather than resulting in widespread structural changes. The trend of the Simpson index is consistent with previous research on the relationship between the gut virus profile and the severity of NAFLD [13]. After correcting for gender using MaAslin2, 185 vOTUs were significantly enriched in non-NAFLD, while 105 were significantly enriched in NAFLD. These vOTUs mainly belonged to the *Siphoviridae*, *Myoviridae*, and *Quimbyviridae*. Host prediction analysis revealed that NAFLD-enriched phages primarily targeted *Bacteroides*, a genus associated with metabolic dysregulation and inflammation. Several of these predicted hosts, including *Bacteroides coprophilus* and *Bacteroides thetaiotaomicron*, have previously been linked to the progressive subtype of NAFLD [30]. In contrast, non-NAFLD-enriched phages were mainly associated with beneficial taxa such as *Faecalibacterium prausnitzii*, *Oscillibacter*, and *Prevotella* [31–33]. These patterns suggest that the NAFLD-associated virome may contribute to disease progression through modulation of key bacterial communities and their functions.

To investigate the functional implications of virome shifts, we integrated serum metabolomics data. Although α-diversity of metabolites showed no group differences, β-diversity analysis revealed significant separation between NAFLD and non-NAFLD subjects. Eight differential metabolites were identified, including nonanoic acid, picolinic acid, and methylsuccinic acid enriched in non-NAFLD, and beta-alanine, isovaleric acid, and DPA enriched in NAFLD. DPA, an ω-3 polyunsaturated fatty acid linked to cardiovascular health and inflammation, ameliorates NAFLD by downregulating SREBP-1c and ChREBP to reduce de novo lipogenesis [34], lowering liver enzyme levels (ALT/AST) [35], activating PPARα to enhance fatty acid oxidation [36], inhibiting NF-κB to reduce pro-inflammatory cytokines [37], and improving lipid profiles [38]. We found that this metabolite was positively correlated with both NAFLD- and non-NAFLD-enriched vOTUs, highlighting the complex, potentially bidirectional interactions between viruses and host metabolism. Although correlation does not imply causation, these associations raise the possibility that the virome contributes to host metabolic phenotypes via viral–bacterial–metabolite networks.

Functional annotation revealed a marked reorganization of viral gene repertoires in the NAFLD gut virome, suggesting altered ecological strategies and host interactions. Several genes associated with DNA recombination and integration, such as *int* (K14059), *recD* (K03581), and *virD4* (K03205), were significantly depleted in NAFLD-enriched viruses. These functions are typically involved in phage integration, horizontal gene transfer, and genome plasticity, implying a reduced capacity for genetic exchange and dynamic adaptation within the NAFLD gut ecosystem [39]. Conversely, *xerD* (K04763), a site-specific recombinase responsible for chromosomal stabilization, was enriched in NAFLD, indicating that viral communities may shift from active recombination toward a more stable, host-associated state [40]. This functional divergence between *int* and *xerD* suggests a transition from mobile, gene-transfer–driven viromes to those characterized by persistent integration and host coexistence, potentially reflecting selective pressures imposed by the altered gut environment in NAFLD. In addition, several stress-response and host-interaction genes were enriched in NAFLD-associated viruses, including *dnaK* (K04043), *hipB* (K15773), *trbJ* (K20266), *luxI* (K22954), and *SMARCAL1* (K14440). These genes are involved in molecular chaperoning, toxin–antitoxin regulation, quorum sensing, and chromatin remodeling, suggesting enhanced viral adaptability and communication with bacterial hosts [41–43]. The increased prevalence of *lysozyme* (K01185) further indicates a potential shift in the balance between lytic and lysogenic cycles, allowing viruses to toggle between dormancy and activation depending on host stress or metabolic conditions [44]. Collectively, these changes point to a functional reprogramming of the gut virome in NAFLD, favoring long-term persistence, metabolic integration, and fine-tuned modulation of bacterial communities. Such adaptations may influence bacterial community stability, immune signaling, and host metabolism, thereby contributing to the pathophysiological landscape of NAFLD.

Importantly, we demonstrated that gut virome signatures have diagnostic potential for NAFLD. The viral model achieved higher predictive accuracy than the bacterial model, suggesting that viral shifts may more directly reflect host metabolic and immune disturbances. Moreover, integrating viral and bacterial features further enhanced classification performance, underscoring the complementary and translational value of cross-domain microbial biomarkers in NAFLD prediction. While these results highlight the virome’s potential as a noninvasive biomarker source, several limitations must be acknowledged. First, this study relied on bulk metagenomic sequencing rather than dedicated viral enrichment protocols, which may have limited the resolution and accuracy of virome characterization and potentially introduced bias in viral detection. Second, dietary and medication information was not available for the analyzed cohort, both of which are strong confounders that could influence metabolomic and microbiome profiles. Third, although the sample size (*n* = 180) is relatively larger than in previous NAFLD virome studies, it still limits the generalizability of our findings and may reduce statistical power to detect low-abundance viral or metabolic features. Fourth, the cross-sectional design precludes causal inference, and longitudinal or interventional studies will be needed to validate the temporal and mechanistic relationships between the gut virome, host metabolism, and NAFLD progression. Finally, potential technical biases—including those arising from DNA extraction, library preparation, and the incompleteness of current viral reference databases—may have affected the comprehensiveness of viral identification. Despite these limitations, our findings provide valuable insights into the role of the gut virome in NAFLD and lay a foundation for future multi-omics and longitudinal investigations.

## Conclusion

In summary, this study provides a comprehensive characterization of the gut virome in NAFLD and its associations with host metabolism. Although overall viral diversity and structure remained relatively stable, specific compositional and functional shifts were evident at the vOTU level. NAFLD-associated viral signatures were dominated by bacteriophages targeting *Bacteroides* and other taxa linked to metabolic dysregulation, whereas non-NAFLD-enriched viruses were mainly associated with beneficial commensals such as *Faecalibacterium* and *Prevotella*. Functional annotation revealed a transition from mobile, recombination-active viromes toward more stable, host-adapted viral communities enriched in stress response and host-interaction genes. Integration with serum metabolomic data further indicated that virus–metabolite correlations may reflect the virome’s influence on host metabolic pathways. Moreover, viral features showed strong diagnostic potential, outperforming bacterial markers, and the combined model achieved the highest predictive accuracy, underscoring the complementary nature of viral and bacterial components. Collectively, these findings highlight the ecological and functional reprogramming of the gut virome in NAFLD and suggest that virome-based biomarkers could serve as promising noninvasive indicators for disease monitoring and prediction. Future longitudinal and mechanistic studies are warranted to clarify the causal role of gut viruses in NAFLD progression and their potential as therapeutic targets.

## Electronic supplementary material

Below is the link to the electronic supplementary material.

Supplementary Material 1

Supplementary Material 2

## Data Availability

The metagenomic datasets analyzed during the current study are available in the NCBI SRA repository under accession numbers PRJNA728908 and PRJNA686835. The serum metabolomic dataset is available in the MetaboLights database under accession ID MTBLS2615.
