# Supplementary material for: Characterization of the gut virome in patients with nonalcoholic fatty liver disease
Source: J Transl Med. 2025 Nov 28;24:6. doi: 10.1186/s12967-025-07443-w (PMC12763994; doi:10.1186/s12967-025-07443-w)
Supplement: Supplementary file 2 — Supplementary Material 2 [file 12967_2025_7443_MOESM2_ESM.docx]

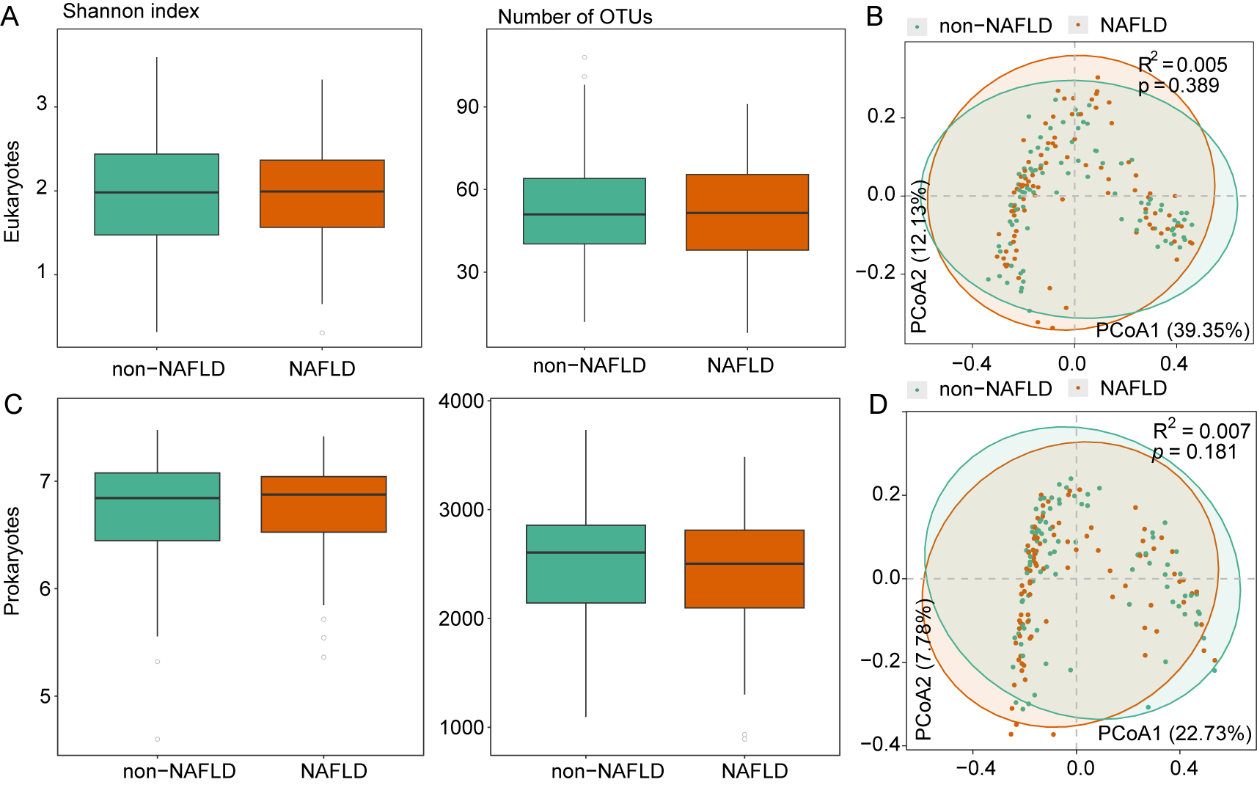
**Figure S1. Comparison of eukaryotic and prokaryotic viral diversity and community structure between NAFLD and non-NAFLD groups.** (A–B) Boxplots showing α-diversity (Shannon index) and richness (number of observed OTUs) of **eukaryotic viruses** in non-NAFLD and NAFLD subjects. (B) Principal coordinate analysis (PCoA) based on Bray–Curtis dissimilarity revealed no significant group separation. Corresponding analyses for **prokaryotic viruses**, including Shannon index and richness (C) and β-diversity via PCoA (D),
